# Textiloma (gossypiboma) mimicking recurrent intracranial abscess

**DOI:** 10.1186/s13104-015-1315-5

**Published:** 2015-08-30

**Authors:** Aykut Akpinar, Necati Ucler, Cengiz Omer Ozdemir

**Affiliations:** Department of Neurosurgery, Adiyaman University Education and Research Hospital, 02200 Adiyaman, Turkey

**Keywords:** Textiloma, Gossypiboma, Brain abscess, Surgery

## Abstract

**Introduction:**

Cranial-retained surgical sponges (gossypiboma or textiloma) are rare incidents and mostly asymptomatic. However, they can be confused with other masses such as a hematoma abscess or tumor. During early stages, some gossypibomas can cause infection or abscess formation.

**Case presentation:**

A 22-year-old Turkish female who had frontal lobe brain surgery to remove an abscess 2 months previously was admitted with complaints of headache and vomiting.

**Conclusion:**

Gossypiboma was confirmed in the patient. Following cranial surgery, gossypiboma should be considered as a differential diagnosis of recurrence of previous surgical operations.

## Background

Although rare, retained surgical sponges can occasionally be found after neurosurgical operations. A “textiloma” describes a mass lesion consisting of a surgical sponge. “Gossypiboma” describes both the mass of sponge and a foreign-body reaction around it [1, 2]. These pathologies can mimic other cranial mass lesions such as hematoma, abscess and tumor. Although well known, their presentation varies with each case because of different reactions of the body. In the literature, only 46 cases of cranial gossypiboma have been reported [1, 3]. However, the real number is thought to be higher, as cases may go unreported because of medicolegal issues. In this reportive present a case of cranial gossypiboma with the clinical presentation, radiological findings, and differential diagnosis of the lesion.

## Case presentation

**Fig. 1:**
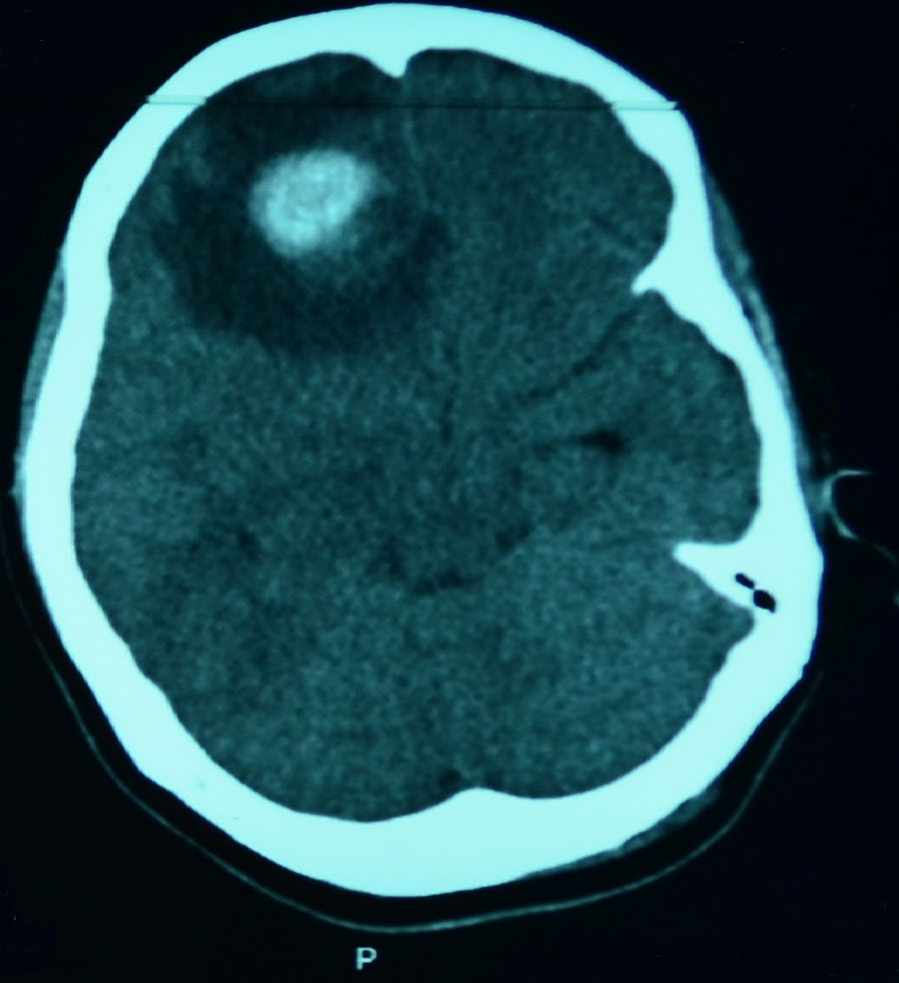
Computed tomography of a mass in the frontal lobe at the site of prior abscess, associated with extensive edema in the surrounding brain region.

**Fig. 2:**
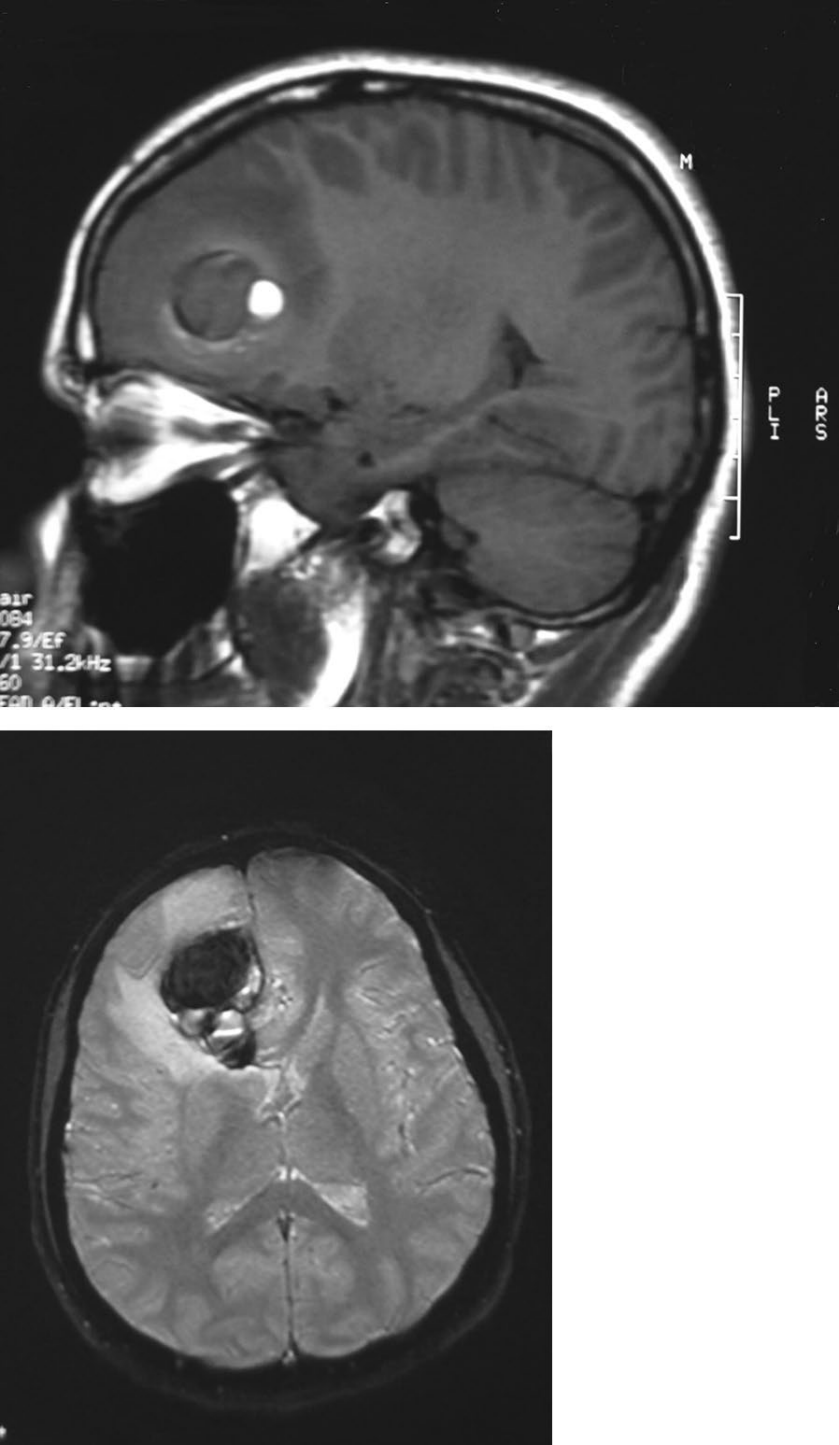
Magnetic resonance imaging showing a new contrast enhancing mass in the frontal lobe at the site of prior abscess.

A 22-year-old Turkish female was admitted with headache and vomiting. She presented with a history of frontal lobe brain abscess which had been operated on in the previous 2 months. The abscess was surgically resected using resorbable hemostatic agents in the operation area. After the initial surgery, third-generation cephalosporin was started and continued for 10 days. The neurologic examination was normal. There was no fever and routine laboratory tests (including complete blood count, erythrocyte sedimentation rate, C-reactive protein and blood biochemistry) were all normal. Microbiologic and pathologic investigations of the abscess revealed no pathogens, only exudative encapsulation. Two months after the operation, the patient admitted to our clinic with increasing frequency of severe headaches. Subsequent computed tomography (CT) and magnetic resonance imaging (MRI) scans revealed a new contrast enhancing mass in the frontal lobe at the site of her prior abscess which was associated with extensive edema in the surrounding brain (Figs. 1, 2). The clinico-radiological differential diagnosis included recurrent abscess, hemorrhage into abscess bed, venous infarction, tumor and radiation necrosis.

Reoperation was performed for resection of the lesion to relieve the mass and provide tissue for a definitive diagnosis so that the appropriate treatment could be administered. The patient was operated on using a bicoronal reincision, and exploration of the frontal lobe area revealed a retained sponge. The sponge was found adherent to the surrounding soft tissue by the newly formed fibrotic tissue, and individual dissection of these fibrotic attachments was required before the abscess was found. The lesion was removed with no intraoperative complications.

**Fig. 3:**
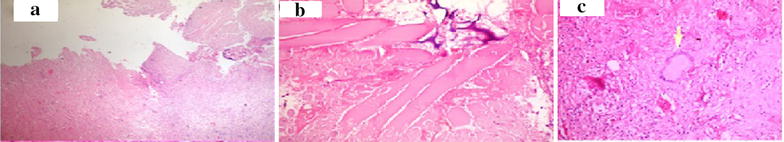
**a** Chronic inflammatory granulation tissue including neuronal tissue and giant cells (Hematoxylin and Eosin Staining, ×4), **b** Fibers surrounded with blood, fibrin histiocytes and inflamatuar cells, including foreign-body type giant cells (Hematoxylin and Eosin Staining, ×20, and **c** Chronic inflamatuar granulation tissue (Hematoxylin and Eosin Staining, ×20).

Histopathology revealed mononuclear clear cell infiltration and fibrosis formation around the retained sponge (Fig. 3a–c). Third generation cephalosporin was continued postoperatively. The patient’s initial symptoms of vomiting and headaches disappeared with no further neurologic deficits, and she was discharged on postoperative day ten without complications.

## Discussion

Textiloma (from Latin “textile”, a woven fabric, plus the suffix “oma”, meaning swelling or tumor), gossypiboma (from Latin “gossypium”, the genus of cotton plants, plus “borna”, a Kiswahili term meaning place of concealment) gauzoma (from surgical gauze) and muslinoma (from muslin) are the historical terms that have been given to foreign-body related inflammatory pseudo-tumors. Specifically, these terms refer to tumors arising from a retained, non-absorbable cotton matrix that is either inadvertently or deliberately left behind during surgery, together with the associated inflammatory reaction.

All classes of resorbable and non-resorbable hemostatic agents may produce textilomas as an allergic response. Textilomas may present with neuroimaging features that mimic recurrent tumor, abscess, and hematoma. In the differential diagnosis of a mass lesion arising after prior intracranial surgery, the possibility of textiloma should be considered along with recurrent tumor, radiation necrosis, and abscess.

Awide variety of synthetic materials may be left in place during intracranial procedures, e.g., silicone coated sheets, which are used as a dura mater substitute for repair of dural defects. Microscopic remnants of cotton gauze of no clinical consequence are often inadvertently left in the surgical field and subsequently identified incidentally on microscopic examination of a specimen obtained at repeat surgery. These agents and other foreign substances that are deliberately introduced to the central nervous system may induce an excessive inflammatory foreign-body reaction [4–6].

Many different kinds of hemostatic agents, absorbable and non-absorbable, are used to control intraoperative bleeding in neurosurgical operations. Non-absorbable materials include various forms of cotton pledgets and synthetic hemostats, which should be removed before surgical closure [7].

In the general surgical literature, the incidence of textiloma is highest following abdominal surgery, followed by orthopedic procedures. Textilomas have been reported in all major anatomic compartments: chest, retroperitoneum, extremities, head and neck [8, 9]. The time interval to clinical presentation ranges from the immediate postoperative period to decades after surgery [10]. Textiloma has been reported significantly less frequently in the neurosurgical literature compared with general surgery. However, it is likely that the incidence is underestimated. The increasing use of MRI monitoring combined with an increasing frequency of repeat surgery in neurosurgery is enabling the opportunity to study these mass lesions.

After surgery, the body can react to foreign bodies such as retained sponges in two ways: (1) exudative tissue reaction which leads to acute abscess formation, and (2) aseptic fibrous tissue reaction, which involves slow adhesion formation such as encapsulation and granuloma formation [11].

Hemostatic agents may produce clinically symptomatic, radiologically apparent mass lesions. When considering a mass lesion arising after intracranial surgery, the differential diagnosis should include textiloma along with recurrent tumor abscess and radiation necrosis.

CT scans can be useful in cases of suspected lesions. However, gossypibomas may not be easily recognized even on CT scans. In our case, the CT scan showed a hyper-dense mass lesion, although it was not diagnostic for the lesion [12, 13]. MRI with intravenous contrast enhancement is known to be the best radiologic investigation modality in these situations [11]. MRI usually shows a well-defined mass with a fibrous capsule that exhibits low signal intensity on T1-weighted images compared with the signal intensity of the brain. High signal intensity in the center with hypo-intense rim on T2-weighted images ABD strong peripheral enhancement in contrast images. However, in our case, MRI demonstrated a mass lesion which was hypo-intense on both T1- and T2-weighted images, with peripheral hyper-intense ring in T1-weighted images and peripheral enhancement in post-contrast images [11]. Accordingly, we believe that despite the importance of MRI in the diagnosis of gossypiboma lesions, the definitive diagnosis must be mainly aided by the high suspicion profile of the physician and the intraoperative findings.

## Conclusions

In patients with history of cranial operation, gossypiboma should always be a consideration in the differential diagnosis of newly found lesions, as it is believed that they are much more common than reported. MRI is the best radiologic modality for the diagnosis. However, no pathognomonic radiologic characteristics are defined for these lesions. For this reason, the definitive diagnosis must be mainly aided by the physician and the intraoperative findings. Moreover, although gossypibomas are rare, their occurrence is still a possibility. Thus it must be remembered that careful inspection of the surgical field before closure is still an important basic rule in surgery.

## Consent

Written informed consent was obtained from the patient for publication of this case report and any accompanying images. A copy of the written consent is available for review by the Editor-in-Chief of this journal.

## Abbreviations

CT: computed tomography
MRI: magnetic resonance imaging
